# Practical Euthanasia Method for Common Sea Stars (*Asterias rubens*) That Allows for High-Quality RNA Sampling

**DOI:** 10.3390/ani11071847

**Published:** 2021-06-22

**Authors:** Sarah J. Wahltinez, Kevin J. Kroll, Elizabeth A. Nunamaker, Nancy D. Denslow, Nicole I. Stacy

**Affiliations:** 1 Department of Comparative, Diagnostic, and Population Medicine, College of Veterinary Medicine, University of Florida, Gainesville, FL 32610, USA; swahltinez@ufl.edu; 2 Department of Physiological Sciences, Center for Environmental and Human Toxicology, College of Veterinary Medicine, University of Florida, Gainesville, FL 32610, USA; krollk@ufl.edu (K.J.K.); ndenslow@ufl.edu (N.D.D.); 3 Animal Care Services, University of Florida, Gainesville, FL 32611, USA; nunamaker@ufl.edu; 4 Department of Biochemistry and Molecular Biology, College of Medicine, University of Florida, Gainesville, FL 32610, USA

**Keywords:** animal welfare, invertebrate, killing, magnesium chloride, sea star, starfish

## Abstract

**Simple Summary:**

Sea stars are iconic marine invertebrates and are important for maintaining the biodiversity in their ecosystems. As humans, we interact with sea stars when they are used as research animals or displayed at public or private aquaria. Molecular research requires fresh tissues that have thus far been considered to be of the best quality if collected without euthanasia. This is the first paper describing a method to euthanize sea stars that still allows for sampling of high-quality tissue that can be used for advanced research. Since it can be difficult to tell if an invertebrate has died, it is important to use a two-step method where the first step makes it non-responsive and the next step ensures it has died. Sea stars were placed in a solution of magnesium chloride until they no longer reacted to having their underside or oral surface tapped. Sea stars did not seemingly find the magnesium chloride solution unpleasant. After they were no longer reactive, the sea stars were immediately sampled. Tissue from their digestive glands was extracted and interpreted to be of adequate quality for molecular research techniques. This study promotes animal welfare while maintaining high-quality tissue sampling for molecular research.

**Abstract:**

Sea stars in research are often lethally sampled without available methodology to render them insensible prior to sampling due to concerns over sufficient sample quality for applied molecular techniques. The objectives of this study were to describe an inexpensive and effective two-step euthanasia method for adult common sea stars (*Asterias rubens*) and to demonstrate that high-quality RNA samples for further use in downstream molecular analyses can be obtained from pyloric ceca of MgCl_2_-immersed sea stars. Adult common sea stars (*n* = 15) were immersed in a 75 g/L magnesium chloride solution until they were no longer reactive to having their oral surface tapped with forceps (mean: 4 min, range 2–7 min), left immersed for an additional minute, and then sampled with sharp scissors. RNA from pyloric ceca (*n* = 10) was isolated using a liquid–liquid method, then samples were treated with DNase and analyzed for evaluation of RNA integrity number (RIN) for assessment of the quantity and purity of intact RNA. Aversive reactions to magnesium chloride solution were not observed and no sea stars regained spontaneous movement or reacted to sampling. The calculated RIN ranged from 7.3–9.8, demonstrating that the combination of animal welfare via the use of anesthesia and sampling for advanced molecular techniques is possible using this low-cost technique.

## 1. Introduction

Sea stars are iconic marine invertebrates and are important for maintaining biodiversity in their ecosystems [1,2,3]. Sea stars are commonly kept under human care for research and for display at public and home aquaria. Sea stars are appealing as research subjects as they possess many unique traits, including impressive regenerative capabilities [4,5]; mutable collagenous tissue capable of rapid nervously mediated changes in tensile strength, which is of great interest to the biomedical industry [6]; adhesives from tube feet [7,8]; and deuterostome-type development similar to vertebrates that makes them a valuable invertebrate model for embryonic development [9,10]. Common sea stars (*Asterias rubens* Linnaeus, 1758) have been used extensively in research as they are easy to collect due to their abundance in the eastern and western northern Atlantic Ocean; they are commonly found from the intertidal zone down to 650 m, and adapt well to life under human care [11,12]. Sea stars may need to be killed due to the deterioration of their health, overpopulation, their status as an invasive or damaging species in some regions, or for research endeavors. The collection of sea star samples in research often requires lethal sampling due to the amount of tissue required for analyses and thus the invasive nature of sample collection. Due to concerns over adequate tissue quality, sea stars used in research are currently often lethally sampled without any prior method rendering them insensible.

The terminology used to describe the act of ending an animal’s life is based on the application of the activity. Euthanasia is used to describe the act of ending the life of an animal using a method that minimizes or eliminates pain and distress. Slaughter is the act of killing animals to harvest them for consumption. Killing is typically used to denote ending an animal’s life in a way that minimizes distress but is not being performed to end an animal’s suffering, as in true euthanasia [13]. The term euthanasia applies to the work presented herein since the goal was to minimize harm to the animal during sampling. For a method to be considered euthanasia, it should achieve rapid unconsciousness and death, minimize stress, and be reliable, reproducible, and irreversible [14]. It is important to note that methods that do not cause rapid death or that result in trauma prior to loss of consciousness cannot be considered euthanasia. Unacceptable methods for invertebrates include removal from water to die by desiccation (i.e., “dewatering”), freezing, or immersion in caustic chemicals (i.e., placing directly in tissue fixative) as a solo or first step [13].

One challenge with ensuring the humane death of sea stars is verification of death. Methods used for verification of death in vertebrate animals include auscultation, electrocardiogram, Doppler ultrasound, and pulse oximetry. However, these techniques are not feasible in echinoderms which do not have pumping structures detectable outside the organism. Methods that can be useful in invertebrate species include a lack of movement, loss of response to stimuli, and flaccidity [13]. In echinoderms, permanent loss of response to stimuli and lack of tube feet suction to substrate can be considered as markers that the animal is insensible and perhaps dead; however, these overlap with anesthesia in echinoderms [15]. To confirm death, a secondary technique should be used that is unsurvivable. The American Veterinary Medical Association Guidelines for the Euthanasia of Animals 2020 Edition recommends a two-step approach for euthanasia of aquatic invertebrates where the first step results in chemical induction of non-responsiveness and is followed by a second step that destroys the brain or major ganglia physically or chemically. The first step can include immersion in magnesium salts, clove oil, eugenol, or ethanol followed by the second step of immersion in 70% alcohol or 10% formalin, or physical methods including pithing, freezing, and boiling [13]. Recommendations for euthanasia of echinoderms include immersion in 7.5% to 8% magnesium chloride (MgCl_2_) or buffered MS-222 (tricaine methanesulfonate) at 1–10 g/L [16]. Immersion in 7–8% MgCl_2_ has been reported in sea stars of various life stages for relaxation prior to sampling for evaluation of gross anatomy and histology [17,18,19,20,21,22,23]; however, the process has not been described in detail and none has been performed for molecular techniques.

Pyloric ceca, also known as digestive glands, are where absorption and storage of nutrients occur in sea stars [24]. Common sea stars undergo a yearly cycle where the pyloric ceca expand and store glycogen during the non-reproductive time in the summer to fall, then lyse to provide nutrients to support gonadal growth over the winter to spring as the gonads are active [25]. Evaluating the RNA in pyloric ceca can inform about toxins [26] and environmental stressors [27] and provide reference genes [28]. To determine RNA quality via the RNA integrity number (RIN), the Agilent 2100 Bioanalyzer (Agilent Technologies, Santa Clara, CA, USA) uses microfluidic technology, combining traditional gel electrophoresis and laser-induced fluorescence detection in the same platform. RIN scores vary from 1–10; samples with a RIN of 10 have no RNA degradation while samples with a RIN of 1 are highly degraded [29,30,31,32,33]. The assessment of RIN is considered an essential step for the evaluation of quantity and purity of RNA before using advanced molecular techniques (e.g., RT-PCR).

The objectives of this study were to (1) describe an inexpensive and effective two-step euthanasia method for adult common sea stars and to (2) demonstrate that high-quality RNA samples useful for molecular diagnostics can be obtained from the pyloric ceca of MgCl_2_-immersed sea stars. The two-step euthanasia technique reported here is immersion in 75 g/L (7.5%) MgCl_2_ until the sea star is non-responsive followed by a physical method of quick dissection.

## 2. Materials and Methods

### 2.1. Sea Star Collection and Husbandry

Sea stars are not currently covered by research oversight guidelines in the United States and thus no Institutional Animal Care and Use Committee (IACUC) review was performed. Sea stars were handled and housed in accordance with guidelines for aquatic species published in the Guide for the Care and Use of Laboratory Animals, 8th edition [34].

Adult common sea stars (*Asterias rubens*) (*n* = 15, 8 females, 7 males) were purchased from a commercial collector (Ocean Resources Incorporated, Sedgwick, ME, USA) in the subtidal zone off the coast of Sedgwick, Maine. Immediately following collection, the sea stars were packaged in perforated plastic bags and a small amount of water in a heavy-weight plastic bag for overnight shipment to the University of Florida. On arrival, sea stars were visually examined, photographed, weighed (g), and contoured diameter (cm) was measured. Contoured diameter was defined as the distance in cm from the longest tip of one ray across the central disc to the tip of the ray directly across using a flexible tape measure across the contoured aboral surface of the sea star at rest. Sea stars were slowly acclimated to temperature and water chemistry over 6 h with ~10% water addition per hour then group housed in a static renewal 454 L (120 gallon) round fiberglass tank in artificial seawater (Instant Ocean, Blacksburg, VA, USA) mixed according to the manufacturer’s specifications. A large air stone connected to compressed air was provided in the center of the tank for aeration. Sea stars were housed in a facility with an air temperature of 25 °C and 16:8 photoperiod automatically maintained with overhead fluorescent lighting on a timer. Sea stars were housed and euthanized in water approximating the temperature of the Atlantic Ocean near the location of sea star collection as reported by buoy I01 of the Northeastern Regional Association of Coastal and Ocean Observing Systems (NERACOOS, http://www.neracoos.org/, accessed on 26 March 2021).

Water quality was tested daily for ammonia, nitrite, and nitrate with a portable colorimeter (DR 900, Hach, Loveland, CO, USA), alkalinity with a drip test kit (Salifert Worldwide, Duiven, Holland), salinity with a refractometer (model RHS-10ATC, Aquatic Eco-Systems Incorporated, Apopoka, FL, USA), and pH and dissolved oxygen with a handheld meter (ProDSS, YSI Incorporated, Yellow Springs, OH, USA). Water temperature was monitored with a max–min thermometer reset daily (Brannan Thermometers & Instrumentation, Cumbria, UK). Water quality was maintained from (min–max) 0.00–0.54 mg/L for ammonia, 0.000–0.027 mg/L for nitrite, 0.0–1.2 mg/L for nitrate, 10.2–10.4 dKh for alkalinity, 33–34 ppt for salinity, 8.09–8.13 for pH, 9.06–10.49 mg/L for dissolved oxygen, and 12.0–12.9 °C for temperature. A 10% water change was performed at least every 72 h with a 25% water change performed as indicated when ammonia or nitrite were above thresholds (ammonia >0.25 mg/L and nitrite >0.1 mg/L).

Sea stars were each fed one frozen clam (Ocean Nutrition, Newark, CA, USA) every 48–72 h. Sea stars were maintained under the laboratory conditions described above and observed daily for two weeks before sampling.

### 2.2. Sea Star Euthanasia and Sampling

Sea star sampling was completed over two days. Each individual sea star was visually examined immediately prior to sampling and was included in the study if it had fed since arrival, had no evidence of visible lesions, and was responsive to external stimuli (e.g., tapping the ambulacral groove with forceps or moving away from conspecifics in the tank). Sea stars were weighed and contoured diameter was measured a second time at time of sampling. A baseline response to stimuli was noted for each individual by tapping the ambulacral groove with a pair of forceps which resulted in tube foot movement and ambulacral groove closure. Sea stars were placed in a 7.5% MgCl_2_ solution of 75 g magnesium chloride hexahydrate (Fisher Scientific, Waltham, MA, USA) dissolved in 1 L de-ionized water chilled to 12 °C. The approximate cost of 75 g of MgCl_2_ is USD 5. Response to tactile stimulation was measured once per minute by quickly removing the sea star from the water and tapping the ambulacral groove with forceps. Sea stars were immediately re-immersed if they were still responsive. Behavior of the sea stars was assessed by continuously observing the sea stars during immersion. Possible aversive reactions to MgCl_2_ based on clinical evaluation, including mucus production, arm curling, stomach eversion, and escape behaviors, were considered for documentation if observed. The time from initial submersion to lack of response to tactile stimulation was recorded. Sea stars were left in the MgCl_2_ solution for 1 min past complete loss of response to tactile stimulation for the purpose of going beyond anesthetic effects of MgCl_2_ immersion. Once sea stars were rendered insensible, they were immediately dissected with sharp scissors at the junction of the aboral and oral body walls. Samples of cardiac stomach, pyloric ceca, gonad, body wall, and tube feet were removed, cut into small pieces (3–4 mm), and immediately flash frozen in liquid nitrogen. The aboral body wall was sampled with a 6 mm punch biopsy (Miltex^®^, Integra, Princeton, NJ, USA). All samples were stored in sterile cryogenic vials with no additives (Corning Incorporated, Corning, NY, USA) at −80 °C until processing. RNA was extracted within 18 days of sampling and stored at −80 °C until analysis. Extracted RNA samples were analyzed within 166 days of sampling, which is well within the time frame of known stability for RNA integrity for up to 15 years if frozen at −80 °C [35].

To determine the sex of each individual sea star, a squash preparation of the gonad was made at the time of sampling. The slides were air dried, stained with Wright–Giemsa (Harleco; EMD Millipore, Billerica, MA, USA), and evaluated via light microscopy.

Samples of artificial seawater from the sea star tank and 75 g/L MgCl_2_ solution were analyzed for ion composition and osmolality. Chloride was measured using Standard Methods 4110 [36] with a Dionex System (ThermoFisher Scientific, Waltham, MA, USA). Cations (Na^+^, Mg^2+^, Ca^2+^, K^+^) were measured using EPA method 6010D [37] with an Ametek SpectroBlue ICP-OES (Spectro Analytical, Kleve, Germany). Osmolality was measured using a vapor pressure osmometer (Wescor Inc., Logan, UT, USA).

### 2.3. RNA Extraction

Ten individuals were randomly chosen with a random number generator for RNA extraction due to costs and time limitations associated with sample preparation and quality analysis. Total RNA was extracted from pyloric ceca with RNA Stat-60 reagent (Tel-test, Friendswood, TX, USA) as previously described [38]. Briefly, 0.1 g of frozen tissue was homogenized in Stat-60 with a tissue grinder. Chloroform was added and the mixture was centrifuged at 20,000× *g* for 15 min at 4 °C. This extraction was repeated with the upper aqueous phase. Following the second centrifugation, total RNA was precipitated overnight with isopropanol. After centrifugation, the pellet was washed with 75% ethanol, air dried, and resuspended in 20 µL of RNAsecure™ (ThermoFisher Scientific, Waltham, MA, USA) following the manufacturer’s protocol to inactivate RNases. The concentration of nucleic acid in each sample was determined using a NanoDrop microvolume spectrophotometer (ThermoFisher Scientific, Waltham, MA, USA). Samples were diluted with RNAsecure™ to bring the RNA concentration to <500 ng/μL, then treated using the TURBO DNA-free™ Kit (ThermoFisher Scientific, Waltham, MA, USA) per the manufacturer’s protocol to remove DNA contamination. Total RNA quality was evaluated using the Agilent 2100 Bioanalyzer (Agilent Technologies, Santa Clara, CA, USA) and RNA 6000 Nano Kit per the manufacturer’s protocol.

### 2.4. Statistical Analyses

Statistical analyses were conducted in R (version 4.0.4, R Foundation for Statistical Computing, Vienna, Austria) using the RStudio integrated development environment (version 1.4.1106, RStudio Public Benefit Corporation, Boston, MA, USA). A significance value (α) of *p* < 0.05 was used to determine statistical significance in all tests. For all analyses, a non-parametric test was performed as the data did not meet the assumptions for parametric analyses. Kruskal–Wallis H-tests were performed using the *Kruskal.test* function in the *stats* package to evaluate differences in size and weight between male and female stars at sampling. A Kruskal–Wallis H-test was also performed to determine if there was a difference in time to lack of response to stimuli between male and female sea stars. To evaluate if size had an impact on time to lack of response to stimuli, sea stars were divided into two groups: above the median diameter (>14.2 cm) and the median diameter and below (≤14.2 cm), then a Kruskal–Wallis H-test was used to determine if there was a difference between the groups.

## 3. Results

### 3.1. Study Animals

On arrival, sea star diameter ranged from 10.2–14.6 cm (mean: 12.5 cm) and weight ranged from 32.9–69.2 g (mean: 51.7 g) (see Table 1). All sea stars were acclimated for 16–17 days. During this time, sea stars appeared clinically normal, behaved normally, and were observed to feed at least once prior to sampling. All 15 sea stars met the inclusion criteria.

At the time of sampling (16–17 days after arrival), sea star diameter ranged from 10.3–16.0 cm (mean: 13.9 cm) and weight ranged from 39.4–77.2 g (mean: 59.7 g). There were seven male and eight female sea stars sampled. There were no significant differences in diameter (*p* = 0.30) or weight (*p* = 0.06) between male and female sea stars at sampling.

### 3.2. Sea Star Euthanasia

No sea stars exhibited aversive reactions to immersion in MgCl_2._ The ambulacral groove of sea stars remained open during immersion, but tube feet consistently lost response to tactile stimulation. The mean time to lack of response to stimuli was 4 min (range: 2–7 min). No sea stars regained spontaneous movement or response to stimuli during sampling. It took approximately 5 min to completely sample each sea star after removal from the MgCl_2_ solution. Male and female sea stars did not show a significant difference in time to lack of response to stimuli (*p* = 0.31). There was no significant difference in time to lack of response to stimuli between larger (diameter >14.2 cm) or smaller (diameter ≤14.2 cm) sea stars (*p* = 0.81).

The ion composition and osmolality of artificial seawater from the sea stars’ tank and MgCl_2_ solution are reported in Table 2.

### 3.3. RNA Quality

**Figure 1:**
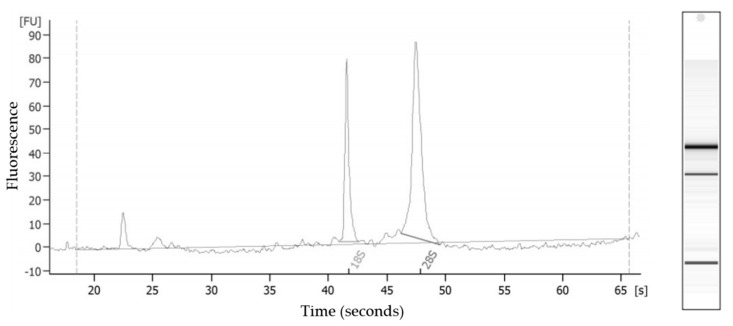
Gel-like image and electropherogram of intact total RNA (RNA integrity number = 9.8) from *Asterias rubens* pyloric ceca analyzed by the Agilent 2100 Bioanalyzer RNA 6000 Nanochip.

Total RNA was extracted from 10 randomly selected pyloric cecum samples. The RIN ranged from 6.8 to 9.8 (mean: 8.7). A gel-like image and electropherogram of intact total RNA are shown in Figure 1. Sampling and RIN data for all sea stars are provided in Table 2.

## 4. Discussion

This study provides proof of concept that the application of animal welfare considerations to molecular research with sea stars is possible. The proposed two-step method of euthanasia with immersion in MgCl_2_ followed by a physical method should quickly render the animal insensible without suffering or distress.

The quick relaxation of sea stars after immersion in MgCl_2_ and lack of adverse reactions or regaining responsiveness indicate that immersion in 75 g/L MgCl_2_ is an acceptable first step in the euthanasia of common sea stars. If samples are not being collected for molecular techniques, sea stars could be left in the MgCl_2_ solution for a longer period of time as immersion for longer than 30 min is recommended in the American Veterinary Medical Association Guidelines for the Euthanasia of Animals 2020 Edition [13]. To minimize stress, sea stars should be euthanized in a solution with a temperature similar to the temperature they are housed at. The osmolality of the tank water and MgCl_2_ solution were similar (809.0 and 802.0 mmol/kg, respectively) which would also minimize potential stress for immersed animals. Unsurprisingly, the concentration of chloride and magnesium were higher in the MgCl_2_ solution than the tank water. Potential stress effects on sea stars resulting from these overt biochemical differences are unknown.

Magnesium chloride has been used to anesthetize or “narcotize” invertebrates for decades [39]. Many species of invertebrates have been anesthetized, which would also serve as the first step of euthanasia, with MgCl_2_, including scallops [40], oysters [41,42,43], queen conch [44], and sea urchins [45]. Magnesium chloride as the first step of euthanasia, followed by destruction of the central nervous system, has been reported for cephalopod mollusks [46]. Immersion in MgCl_2_ (142.25 g/L) was effective as the sole step for euthanasia in jellyfish [47]. To euthanize sea stars, immersion in MgCl_2_ has been followed by immersion in 5–10% buffered formalin [20,23]. A similar euthanasia technique of immersion in MgCl_2_ followed by dissection has been performed; however, those samples were then placed into fixative for histology [17,21], put into 1% glutaraldehyde to photograph and measure [19], or weighed to determine seasonal cycles in pyloric ceca and gonads [22]. One of the limitations of this study is that an air bubbler was not used in the MgCl_2_ tank. This likely did not have an effect on the sea stars in this study as they were immersed for a short time and sea stars can tolerate hypoxia. The median sublethal oxygen concentration for echinoderms was reported as 1.22 mg O_2_/L [48] and they can survive for several days at 0.2 mg O_2_/L [49]. However, it is recommended to use an air bubbler for all anesthetic and euthanasia solutions for aquatic animals since hypoxia is not an approved euthanasia method [13].

The mechanism of MgCl_2_ in invertebrates is unknown, although it is thought to act on the post-synaptic membrane at the neuromuscular junction [50]. Increased magnesium inhibits acetylcholine, inhibiting post-synaptic potentials and causing decreased muscle fiber membrane excitability [51]. In vertebrates, MgCl_2_ enhances the effects of non-depolarizing muscle relaxants [52]. Historically, there has been a debate on if MgCl_2_ blocks nerve transmission and neurotransmitter release or if it acts solely as a neuromuscular blocking agent [53]. Recent literature has shown that the former appears to be true; cephalopod mollusks immersed in MgCl_2_ showed loss of both afferent and efferent signals which indicates the animals had true anesthesia and would not feel subsequent procedures or sampling [46]. There have been no investigations on the impact of MgCl_2_ on neurotransmission in adult echinoderms to date.

The second step in a two-step method of euthanasia should ensure a rapid, non-reversible death. The secondary method described herein is a physical method of quick dissection. Other acceptable secondary methods include immersion in 70% alcohol, immersion in 10% formalin or physical methods including pithing, freezing, and boiling [13]. The secondary step can be altered depending on the ultimate goal for sample analyses. For example, if histology is desired, placement in 10% formalin as a second step would facilitate both killing the sea star and sample fixation. However, formalin results in crosslinks which chemically modify RNA and preclude isolation and evaluation of RNA [54]. When possible, the second step in a two-step method of euthanasia should result in destruction of the central nervous system. The echinoderm circumoral nerve ring and radial nerve cords are considered the central nervous system [55,56]. However, there is no evidence that these are coordinating structures, rather the circumoral nerve ring around the mouth appears to provide connections between radial nerve cords which extend down the arms of sea stars [57]. Common sea stars do not have nervous tissue that is visible with the naked eye which precludes the ability to pith them. However, our thorough sampling method presumptively resulted in the rapid destruction of the majority of nervous tissue. In invertebrate species with a visible nervous system center (e.g., decapod crustaceans and cephalopod mollusks), the authors recommend rapid destruction of the central nervous system in the secondary step to ensure nervous transmission ceases quickly.

When assessing methods appropriate for euthanasia of invertebrates, it is important to bear in mind that invertebrates are a highly diverse group of animals which make up more than 95% of the species on Earth [58]. Aquatic invertebrates have a wide range of nervous system complexity, from sponges which have no true nervous tissue but are able to respond to stimuli [59] to cephalopod mollusks which are arguably the most complex and have more than half a billion neurons [60]. One of the most common euthanasia methods in use is an overdose of anesthetics. Anesthetics commonly used with aquatic invertebrates include buffered MS-222 (tricaine methanesulfonate), ethanol and MgCl_2_ [61]. However, it is important to ensure the animal does not find the anesthetic immersion aversive. Recent evidence has shown that some animals, including zebrafish and sea snails, may find immersion in MS-222, even buffered, to be noxious [62,63]. This appears to be true for sea stars as well; small spine sea stars (*Echinaster spinulosus*) displayed behavioral indicators that they found immersion in 0.8 g/L buffered MS-222 aversive, including mucus production, arm curling, and stomach eversion (unpublished data).

Invertebrate welfare has received increasing attention following the inclusion of cephalopod mollusks in the European Union Directive 2010/63/EU in 2010 [64,65]. At the forefront of the conversation is the ability of invertebrates to feel pain [66,67]. Although there is mounting evidence for invertebrate pain perception, it is not unanimously agreed upon in the scientific and veterinary communities. For the purpose of considering animal welfare, the authors recommend following the precautionary principle and ensuring that care is taken to prevent potential pain and suffering in invertebrate species during euthanasia and sampling processes. To maintain the social license to use invertebrates in research, the ethics must also be considered. The two largest ethical dilemmas identified in invertebrate research by Drinkwater et al. [68] are collection of individual animals and euthanasia practices. The current study represents a small step towards greater inclusion of additional invertebrate species into consideration in animal welfare regulations by building up data on humane euthanasia practices.

The Agilent Bioanalyzer reports RNA quality as a RIN score on a scale from highly degraded (RIN = 1) to completely intact (RIN = 10) [29,30,31,32,33]. There is currently no consensus on a RIN cutoff for sufficient RNA quality in a given sample, with proposed numbers ranging from 3.95 [69] to 8 [70]. The most commonly reported acceptable RIN is 5 or greater. This number has been found to be acceptable as a basis for downstream applications such RT-PCR [71,72] and gene expression analysis [73]. However, the RIN was not a good predictor of microarray performance [74]. All samples in this study had a minimum RIN > 5 (minimum 6.8) which indicates these samples would likely be useful for many analyses. Pyloric ceca were chosen as the tissue for extraction as they were the most abundant tissue collected and is a common target for RNA isolation in sea stars [26,27,75]. We were able to extract high-quality RNA (RIN > 8) from eight out of 10 pyloric cecum samples. The two samples with RIN < 8 initially did not have a RIN calculated by the Agilent Bioanalyzer due to critical errors in the instrument. The anomaly threshold for these errors were manually adjusted per the Agilent Bioanalyzer Troubleshooting Guide [76] and a RIN was calculated. It is unlikely that immersion in the MgCl_2_ solution resulted in a lower RIN since these two individuals were immersed for 3 and 4 min, which was below and at the median time of 4 min, respectively. Factors which could have influenced the RIN include pre-analytical variables such as sampling time and method as well as RNA extraction technique. A single person performed all RNA extractions to minimize variation, however, errors can occur in the extraction process. One limitation of this study is that the isolated RNA was not used for downstream analyses, such as qPCR, microarrays, or transcriptomics, for further assessment of RNA quality. However, given the high quality of RNA based on RIN, it can be presumed that these samples would be useful for a variety of molecular analyses. Samples from sea stars immersed in MgCl_2_ may also be useful for techniques not involving nucleotides, such as metabolomics. Jellyfish euthanized in MgCl_2_ provided useful samples for NMR-based metabolomics [47]. To further determine if MgCl_2_ immersion had an effect on RNA quality, paired molecular tests (i.e., qPCR or transcriptomics) between immersed and non-immersed sea stars should be performed. Since RIN values provide an assessment of total RNA integrity, further studies will be necessary to address potential effects of anesthetic procedures on gene expression profiles.

## 5. Conclusions

The two-step euthanasia method described herein was quick, inexpensive, and effective, and allowed for the collection of high-quality RNA samples. The authors recommend immersion of sea stars in 75 g/L MgCl_2_ as the first step of euthanasia as sea stars were quickly rendered insensible. The secondary step of euthanasia may vary depending on the goals of euthanasia and sampling; quick dissection with sharp scissors, as described here, was a physical method that allowed for sample collection and ensured death of the individual. This method provides a practical way to improve the animal welfare of a sea star species commonly used in research settings and provides a springboard for future studies. Future investigations could evaluate the efficacy of higher and lower MgCl_2_ concentrations and the optimal immersion time.

## Figures and Tables

**Table 1:** Data for *Asterias rubens* (*n* = 15) at time of sampling, including diameter (cm), wet weight (g), sex, time in 75 g/L magnesium chloride solution until non-reactive to stimuli (minutes), and RNA integrity number (RIN, *n* = 10) of RNA isolated from pyloric ceca. NP= not performed.

ID	Diameter (cm)	Weight (g)	Sex	Time (min)	RIN
1	14.6	77.2	F	4	NP
2	10.3	43.5	F	3	7.3
3	13.1	55.8	M	6	NP
4	11.0	39.4	M	2	NP
5	15.0	65.9	M	3	9.8
6	15.4	74.5	F	3	9.5
7	15.5	71.3	F	4	9.0
8	14.2	50.7	F	3	9.3
9	14.7	64.8	F	2	NP
10	13.3	53.8	M	3	8.8
11	13.5	66.1	F	3	8.4
12	16.0	64.8	M	6	NP
13	14.1	55.4	M	7	9.3
14	15.1	71.3	F	5	8.3
15	12.1	41.3	M	4	6.8

**Table 2:** Ion concentrations and osmolality for artificial seawater from sea star housing (tank water) and magnesium chloride solution (75 g/L MgCl_2_).

Sample	Cl^−^ (mg/L)	Na^+^ (mg/L)	Mg^2+^ (mg/L)	Ca^2+^ (mg/L)	K^+^ (mg/L)	Osmolality (mmol/kg)
Tank Water	15,017.0	9224.0	1496.0	381.0	466.0	809.0
75 g/L MgCl_2_	18,527.0	49.0	6557.0	165.0	23.0	802.0

## Data Availability

The data presented in this study are available on request from the corresponding author.
